# Demographics, distribution, ownership and naming patterns of pets presented to a mobile clinic for sterilisation in Namibia

**DOI:** 10.4102/jsava.v91i0.2006

**Published:** 2020-04-29

**Authors:** Ian J.M. Baines, Sharon Baines, Borden Mushonga, Brighton Gorejena, Priscilla Mbiri, Alaster Samkange, Erick Kandiwa, Oscar Madzingira

**Affiliations:** 1 School of Veterinary Medicine, Faculty of Agriculture and Natural Resources, University of Namibia, Windhoek, Namibia

**Keywords:** Namibia, mobile clinic, sterilisation, dogs and cat, ownership

## Abstract

This study analysed the demographics, spatial distribution, ownership and naming patterns of dogs and cats presented to the University of Namibia’s veterinary mobile clinic for sterilisation from small underserved towns around Namibia. The proportional distribution of pets was determined based on species, sex, age, owner gender, town of origin and naming categories. Overall, 84.4% (*n* = 2909) of the animals presented for sterilisation were dogs and the remainder were cats (15.6%, *n* = 539). Of the dogs presented for sterilisation, 51.9% (*n* = 1509) were male and 48.1% (*n* = 1400) were female. In cats, 51.4% (*n* = 277) were male, whilst 48.6% (*n* = 262) were female. Overall, the majority of pets (68.2%) were presented for sterilisation from urban areas than rural areas (31.8%). About 49.8% of men and 24.2% of women that presented pets for sterilisation came from urban areas, whilst 20.1% of the women and 11.7% of the men that presented pets for sterilisation were from rural areas. Of all the pets presented for sterilisation, the majority were male-owned (64%, *n* = 2206). Pets were mainly presented for sterilisation at < 2 years (41.1%), 2 to < 4 years (32.4%) and 4 to < 6 years (15.4%). The naming of pets was mainly after people (42.4%), circumstances (20.6%) and appearance (15.5%). This community engagement exercise yielded valuable demographic data indicating that pet origin, sex and species and owner gender were important factors in determining the voluntary presentation of pets for sterilisation in the study area.

## Introduction

The Society for the Prevention of Cruelty to Animals (SPCA), Have a Heart Foundation, and the Cat Protection Society are some of the animal welfare societies in Namibia whose main objective is to promote and safeguard the welfare of animals in the country. The issue of overpopulation of dogs and cats is a constant irritant to society in light of its public health, socio-economic and animal welfare implications all over the world (American Society for the Prevention of Cruelty to Animals [ASPCA] 2011; Bauer et al. 2016; Faver 2009; Fennell 1999; Hassan & Fromsa, 2017; Ortega-Pacheco et al. 2007). In Namibia, private clinics and public institutions work together with animal welfare organisations to undertake sterilisation campaigns of animals belonging to underserved communities that cannot afford the cost of sterilisation. A case in point is the BAINES VETCARE Mobile Clinic that partnered with the University of Namibia’s (UNAM’s) School of Veterinary Medicine (SoVM) in sterilisation campaigns of dogs and cats from underserved communities of Namibia as part of veterinary students’ training.

The human population of Namibia is estimated at 2 643 075 with an average household size of 3.9 individuals (Namibia Statistics Agency 2016). The population of dogs and cats in Namibia is currently estimated to be between 158 282 and 167 037 owned dogs and 56 149 owned cats (Government of the Republic of Namibia 2017; Namibia Statistics Agency 2015a). There is, therefore, an average of one dog for every 16 people or one dog for every four households and one cat for every 35 people or one cat for every 12 households. Literature abounds with demographics of dog populations throughout the world (Acosta-Jamett et al. 2010; Gsell et al. 2012; Jackman & Rowan 2007; Ortega-Pacheco et al. 2007). Some publications deal with demographics of both dogs and cats (ASPCA 2011; British Small Animal Veterinary Association [BSAVA] 2013; British Veterinary Association [BVA] 2018; Clancy & Rowan 2003; Downes et al. 2015; Odendaal 1994; Kass, Johnson & Weng 2013), whilst fewer publications deal with the demographics of cat population alone (Carvelli, Lacoponi & Scaramozzino 2016; Johnston, Szczepanski & McDonagh 2017; Murray et al. 2015).

In recent times, there have been serious global concerns about pet overpopulation with a resultant increase in the number of animals euthanised by animal shelters (Bauer et al. 2016; Fennell 1999; Meredith et al. 2018). Debates continue to explore the relative contribution of uncontrolled pet animal breeding to the increased annual euthanasia of animals. Studies have blamed the breakdown of human–animal bond, and not increased reproduction, for the annual increase in pet euthanasia, insinuating that the increasing efforts to reduce breeding might actually be misinformed (Clancy & Rowan 2003; Kass, Johnson & Weng 2013). Studies have shown that puppies are not in the majority of animals that are euthanised each year, confirming that reproduction is not a major contributor to increased euthanasia of animals. In a recent study conducted at the Windhoek Animal Shelter, it was shown that only 12% of the animals that were euthanised during a 6-month period were puppies (1st Author, unpublished data). There is no arguing that failure to sterilise pets inadvertently leads to pet overpopulation, even though it is not a major cause of overpopulation (Downes et al. 2015). Although the sterilisation of pets not required for breeding may be considered controversial or even illegal in some countries (Greenfield, Johnson & Schaeffer 2004; Howe 2015; Palmer, Corr, & Sandøe 2012), the Namibian legislation does not prohibit sterilisation of pets for the purpose of controlling the pet population (prior to the Animals Protection Act 1962). A recent issue of *The Namibian* (2013) newspaper published an article advocating for mandatory pet sterilisation, citing animal welfare organisations’ support for neutering and spaying of dogs and cats.

It has been reported that neutering and spaying of male and female dogs and cats reduces reproductive diseases, and consequently undesirable breeding-related behaviours, thereby strengthening bonds between pets and their owners (De Cramer & May 2015; Reichler 2009; Root 2012; Smith 2014). It has also been suggested that sterilisation may increase pet longevity (Smith 2014). Sterilisation of pets, however, is not without its shortcomings (Howe 2015; Scott et al. 2002; Spain 2006; Palmer et al. 2012; Root 2012). Urinary, prostate and some orthopaedic problems have been frequently associated with spaying (Bryan et al. 2007; Palmer et al. 2012; Reichler 2009; Root 2012).

The dog population has received peripheral mention in the publications dealing with domestic canine and feline conditions in Namibia and elsewhere in Africa (Gowtage-Sequeira et al. 2009; Gsell et al. 2012; Haimbodi, Mavenyengwa & Noden 2014). Although total numbers of these pets have been reported in animal censuses and annual reports (Government of the Republic of Namibia 2017; Namibia Statistics Agency 2015b), Namibian pet demographics and ownership patterns have not been reported previously. Whilst population numbers and demographics of dogs and cats owned by the more affluent segments of society (who can afford veterinary care) are obtainable from veterinarians, the same cannot be said for pets from underserved communities. It has been inferred that this population of pets harbours and transmits diseases to owned pets, feral animals, wildlife, livestock as well as human population (Ortega-Pacheco et al. 2007).

According to literature, names of inanimate and animate objects, including individual pets, have their roots in linguistics (Kido 2017, 2018) and psychological discourse or semiosis (Borkfelt 2011; Olaosun & Arua 2012). Understanding the basis on which pets of underserved communities of Namibia are named may shed more light on the social, cultural and economic importance of pets in these communities.

The objective of this study was to analyse the demographics, distribution, ownership and naming patterns of pets from the underserved communities of Namibia which were presented to UNAM’s mobile clinic for sterilisation over a 2-year period.

## Research methods and design

The UNAM’s animal mobile clinic embarked on a country-wide programme to sterilise dogs and cats, strategically targeting 26 underserved communities in the Erongo, Karas, Kavango-East, Khomas, Kunene, Omaheke, Otjozondjupa and Zambezi regions of Namibia.

Dogs and cats residing in 26 underserved communities around Namibia constituted the study population. The study animals were pets that were voluntarily presented for sterilisation.

The clinic was equipped with drugs, surgical equipment, a surgical space and a recovery area. Communities were notified 2–3 weeks prior to the impending visits through alerts and bulletins by local authorities and through local radio stations. Animals presenting with health problems were treated on site. For each animal visiting the clinic, the pet name, date of birth, sex of animal, name and gender of owner were recorded. Naming categories were designed to explain the rationale behind each pet name (Table 1). Standard anaesthetic protocols for dogs and cats were strictly adhered to during the sterilisation procedures.

**TABLE 1:** Detailed explanation of designated pet naming categories.

Naming category	Detailed explanation
Animal	Pet named after an animal (e.g. bear, lion and wolf)
Appearance	Named based on coat colour, hair type, body shape, body markings and general patterns
Circumstances	Name based on event, circumstances or expectations of significance to owner
Companion	Pet given name elevating it as a family member or beloved companion
Dog or cat name	Pet given a generic dog name or cat name (e.g. Buster, Bokkie and Spike)
Person	Named after a person
Icon	Named after an iconic movie or historical character
None	Named simply as doggie, puppy, kitty, etc.

Data were captured from the mobile clinic register onto a Microsoft Excel 2013 spreadsheet. Pivot tables were subsequently used to determine the proportional distribution of animals based on species, sex, age at sterilisation, town of origin, gender of owner and naming category. The Z test was used for comparison of proportions of presented male and female pets based on the gender of the owners and comparison of proportional naming categories for male and female dogs and cats.

### Ethical considerations

The data used in this study were obtained and used with permission from the University of Namibia. No personal information that could identify the participants was used in the study.

## Results

The overall proportion of dogs that were encountered in this study was greater than that of cats (84.4% and 15.6%, respectively; *N* = 3448) (Table 2). About 51.9% (*n* = 1509) of dogs that were brought in for sterilisation were male dogs, whilst the rest (48.1%, *n* = 1400) were female dogs. In case of cats brought for sterilisation, about 51.4% (*n* = 277) were male cats, whilst the rest (48.6%, *n* = 262) were female cats. Of all the pets presented for sterilisation, the majority (64%, *n* = 2206) were owned by men, and the rest (36%, *n* = 1242) were owned by women.

**TABLE 2:** Overall categorical proportions of pets brought in for sterilisation.

Category	Dogs						Cats						Overall total	
	Male		Female		Subtotal		Male		Female		Subtotal			
	*n*	%	*n*	%	*n*	%	*n*	%	*n*	%	*n*	%	*n*	%
Male-owned	1022	29.6	890	25.8	1912	55.5	162	4.7	132	3.8	294	8.5	2206	64.0
Female-owned	487	14.1	510	14.8	997	28.9	115	3.3	130	3.8	245	7.1	1242	36.0
Total	1509	43.8	1400	40.6	2909	84.4	277	8.0	262	7.6	539	15.6	3448	100.0

**FIGURE 1:**
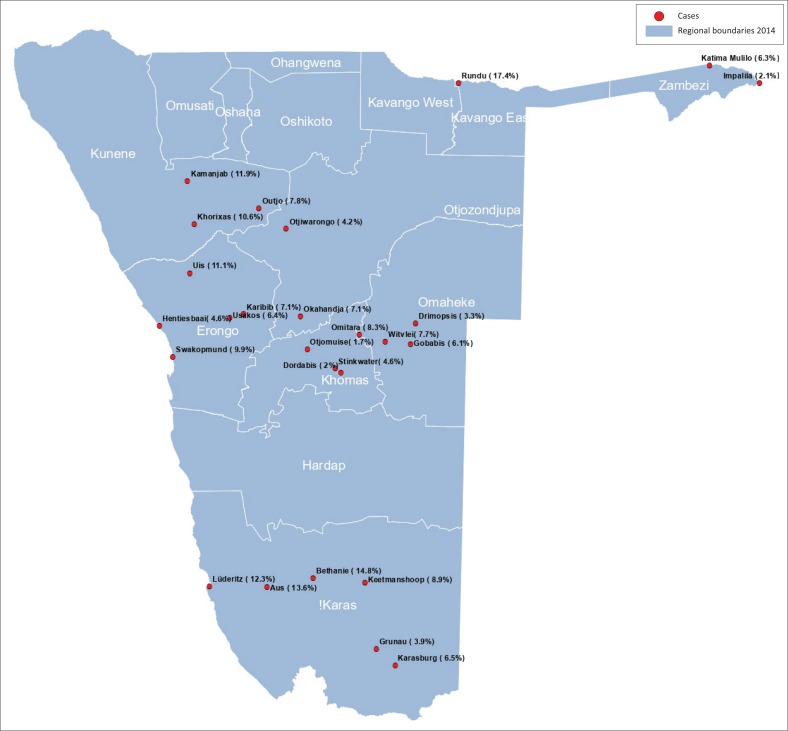
Map of Namibia showing the proportions of pets presented for sterilisation at 26 rural and urban stations.

Overall, 68.2% of the animals presented for sterilisation originated from urban areas, whilst the rest (31.8%) were from rural areas (Figure 1; Table 3). The greatest proportions of animals were presented from urban areas, that is, from Rundu (17.4%), Luderitz (12.3%), Swakopmund (9.9%) and Keetmanshoop (8.9%), whilst the least proportions were presented from Otjomuise (1.7%), Dordabis (2.0%) and Impalila (2.1%). In rural areas, the proportions of male-owned cats from Bethanie (11.1%) and Dordabis (6.9%) were significantly greater than the proportions of female-owned cats (none from both areas; *p* < 0.05). In Omitara, however, the proportion of female-owned cats (20.6%) was significantly greater than that of male-owned cats (8.3%, *p* < 0.05). In urban areas, the proportion of female-owned cats in Gobabis (8.9%) was significantly greater than that of male-owned cats (1.3%, *p* < 0.05). In Swakopmund, the proportion of male-owned dogs (14.2%) was significantly greater than that of female-owned dogs (4.1%, *p* < 0.05). There were no significant differences in gender-based ownership of dogs and cats in the rest of rural and urban areas.

**TABLE 3:** Proportions of species of pets brought in for sterilisation based on towns and gender of pet owners.

Town	Female-owned				Male-owned				Total	
	Cats		Dogs		Cats		Dogs			
	*n*	%	*n*	%	*n*	%	*n*	%	*n*	%
**Rural**										
Aus	13	20.6	38	11.1	8	11.1	90	14.5	149	13.6
Bethanie	0	0.0*	59	17.3	8	11.1*	95	15.3	162	14.8
Dordabis	0	0.0*	5	1.5	5	6.9*	12	1.9	22	2.0
Drimiopsis	3	4.8	13	3.8	4	5.6	16	2.6	36	3.3
Grunau	4	6.3	17	5.0	4	5.6	18	2.9	43	3.9
Impalila	0	0.0	14	4.1	1	1.4	8	1.3	23	2.1
Kamanjab	5	7.9	57	16.7	4	5.6	64	10.3	130	11.9
Katima Mulilo	0	0.0	25	7.3	1	1.4	43	6.9	69	6.3
Khorixas	12	19.0	37	10.9	12	16.7	55	8.9	116	10.6
Omitara	13	20.6*	18	5.3	6	8.3*	54	8.7	91	8.3
Stinkwater	4	6.3	4	1.2	4	5.6	38	6.1	50	4.6
Uis	2	3.2	36	10.6	1	1.4	83	13.4	122	11.1
Witvlei	7	11.1	18	5.3	14	19.4	45	7.2	84	7.7
**Subtotal**	**63**	**100.0**	**341**	**100.0**	**72**	**100.0**	**621**	**100.0**	**1097**	**100.0**
**Urban**										
Gobabis	16	8.9*	46	6.9	3	1.3*	78	6.1	143	6.1
Henties bay	5	2.8	54	8.1	7	3.1	43	3.3	109	4.6
Karasburg	13	7.2	53	8.0	12	5.4	75	5.8	153	6.5
Karibib	13	7.2	63	9.5	16	7.2	75	5.8	167	7.1
Keetmanshoop	19	10.6	82	12.4	14	6.3	94	7.3	209	8.9
Luderitz	12	6.7	105	15.8	18	8.1	154	12.0	289	12.3
Okahandja	15	8.3	34	5.1	27	12.1	90	7.0	166	7.1
Otjiwarongo	13	7.2	19	2.9	5	2.2	62	4.8	99	4.2
Otjomuise	4	2.2	4	0.6	7	3.1	24	1.9	39	1.7
Outjo	12	6.7	40	6.0	25	11.2	106	8.2	183	7.8
Rundu	34	18.9	72	10.9	55	24.7	249	19.4	410	17.4
Swakopmund	4	2.2	27	4.1*	19	8.5	183	14.2*	233	9.9
Usakos	20	11.1	64	9.7	15	6.7	52	4.0	151	6.4
**Subtotal**	**180**	**100.0**	**663**	**100.0**	**223**	**100.0**	**1285**	**100.0**	**2351**	**100.0**
**Grand total**	**243**	**7.0**	**1004**	**29.1**	**295**	**8.6**	**1906**	**55.3**	**3448**	**100.0**

* , Proportions within the same row were significantly different as *p* < 0.05.

Pets were mainly presented for sterilisation at < 2 years of age (41.1%), 2 to < 4 years (32.4%) and 4 to < 6 years (15.4%) (Table 4). Cats were mainly presented for sterilisation at < 2 years of age (56.4%), 2 to < 4 years (31.2%) and 4 to < 6 years (8.9%). In case of dogs, animals were mainly presented for sterilisation at < 2 years of age (38%), 2 to < 4 years (32.6%) and 4 to < 6 years (16.7%).

**TABLE 4:** The overall age at sterilisation according to species of pet.

Age of sterilisation category	Cats		Dogs		Total	
	*n*	%	*n*	%	*n*	%
< 2 years	246	56.4	820	38.0	1066	41.1
2 to < 4 years	136	31.2	704	32.6	840	32.4
4 to < 6 years	39	8.9	360	16.7	399	15.4
6 to < 8 years	8	1.8	159	7.4	167	6.4
≥ 8 years	7	1.6	114	5.3	121	4.7
**Total**	**539**	**100.0**	**2909**	**100.0**	**3448**	**100.0**

The naming of pets was mainly based on people’s names (42.4%), circumstances (20.6%) and physical appearance (15.5%) (Table 5). A greater proportion of male dogs (49.6%) and female cats (45.8%) were named after people. The greatest proportion of pets with no names were female (14.4%) and male cats (10.1%). The proportion of female cats named after animals (8.8%) was significantly greater than that of female dogs named after animals (1.8%, *p* < 0.05). The proportion of male dogs named after a dog name (9.6%) was significantly greater than that of male cats named after a cat name (0.8%, *p* < 0.05). The proportion of male cats named after people (51%) was significantly greater than that of male dogs named after people (32.5%, *p* < 0.05). There were no significant differences in the proportions of male dogs and male cats or female dogs and female cats in the rest of the naming categories.

**TABLE 5:** Proportions of naming categories according to species and sex of pets.

Category	Dogs						Cats						Total	
	Male		Female		Subtotal		Male		Female		Subtotal			
	*n*	%	*n*	%	*n*	%	*n*	%	*n*	%	*n*	%	*n*	%
Animal	76	5.1	25	1.8*	101	3.5	10	4.0	20	8.8*	30	6.3	131	3.9
Appearance	280	18.9	172	12.5	452	15.8	36	14.5	27	11.9	63	13.3	515	15.5
Circumstances	359	24.2	228	16.6	587	20.5	52	20.9	49	21.7	101	21.3	688	20.6
Companion	64	4.3	182	13.2	246	8.6	13	5.2	15	6.6	28	5.9	274	8.2
Dog/cat name	142	9.6*	35	2.5	177	6.2	2	0.8*	2	0.9	4	0.8	181	5.4
Person	482	32.5*	695	50.5	1 177	41.2	127	51.0*	108	47.8	235	49.5	1412	42.4
Icon	78	5.3	39	2.8	117	4.1	9	3.6	5	2.2	14	2.9	131	3.9
**Total**	**1481**	**100.0**	**1376**	**100.0**	**2 857**	**100.0**	**249**	**100.0**	**226**	**100.0**	**475**	**100.0**	**3332**	**100.0**

* , Proportions within the same row were significantly different as *p* < 0.05.

## Discussion

To the best of our knowledge, the current study is the first to focus on the species, population numbers, demographics, ownership and naming patterns for pets from the underserved communities. Statistics obtained from this study, however, may present a keyhole view into the happenings of Namibia’s greater pet population. Thus, this information must be interpreted with caution as it relates only to what is happening to pets volunteered for sterilisation by owners from the underserved communities. However, it is still possible to draw cautious inferences from these underserved communities’ pets about Namibia’s general pet population. Understanding the demographic data obtained from this study could be useful for Namibian veterinary authorities and animal welfare organisations as baseline data for the design and implementation of programmes for the control of pet population and public health purposes (Bögel 1990; Carvelli et al. 2016). The high proportion of dogs may assist in indicating quantities and types of materials that mobile clinics should carry when they visit underserved communities. The study shows that mobile clinics are better off carrying more materials used for dogs than for cats.

Significantly more dogs than cats (84.4% and 15.6%, respectively, *N* = 3448) were presented for sterilisation to the mobile clinic over a period of 2 years. It is, however, noteworthy that these figures should not be considered as the total pet population estimates in any region or in the country. The figures provided in this study were only for pets presented for sterilisation. It is possible that some owners from the underserved communities did not bring their pets for sterilisation. In addition, owned pets from the affluent society and feral dogs and cats were not accounted for in this study. Studies have revealed that relative proportions of pet species presented for sterilisation depended on factors such as literacy status, ethnic background, cultural beliefs, religious affiliation, rural or urban region, and personal economic factors of pet owners (Root 2012). It goes without saying that most of the people often choose to keep cats rather than dogs because of the lower expenses associated with cats. Cats can literally take care of themselves whereas dogs have been found to struggle when they do not have someone to feed them. In addition, cats tend to provide better companionship than dogs as they spend most of their time indoors.

The higher number of dogs than cats presented for sterilisation is contrary to the results of other studies focusing on all owned pets from the United States, which showed that there were roughly equal number of cats and dogs (Clancy & Rowan 2003; Kass et al. 2013). Another contrasting report from the same country, however, indicated that there were actually more cats than dogs (Burns 2019). Furthermore, Johnston et al. (2017) reported that there were roughly two cats per household in Australia and New Zealand. Other studies have also reported presentation of more cats for sterilisation (Faver 2009; Root 2012; Trevejo, Yang & Lund 2011). A study from the mid-1990s in South Africa has shown that there were 80.2% dogs and 19.8% cats (Odendaal 1994), a trend similar to the one observed in this study. However, it is not clear from the results of this study, whether the higher number of dogs than cats is a result of the underserved communities of Namibia placing higher value in dogs than cats or that dogs were easily obtainable.

Overall, there were more male (51.8%) than female animals (48.2%) presented for sterilisation. It has been observed that neutering male animals has a greater impact on population control as one male animal has a potential to populate the whole neighbourhood in a short space of time. The results of this study are contradictory to those in which more female than male dogs were presented for sterilisation (New et al. 2000; Root 2012; Trevejo et al. 2011). This male-biased gender ratio is attributed to the selection of male dogs as pets, perhaps because of the perception that male dogs make better guard dogs than female dogs and to avoid the nuisance of owning a bitch in oestrus or having to deal with unwanted puppies. A bitch in oestrus tends to be a nuisance in the neighbourhood because she attracts groups of intact male dogs (Totton et al. 2010). The higher proportion of male dogs presented for sterilisation in this study may also be related to individual cultural beliefs of this segment of Namibian society (Downes et al. 2015).

It has been suggested that owners are more likely to present their pets for sterilisation if they consider them as companions rather than working animals. The information about the way the underserved community views their pets was not extracted in this study. It has also been suggested that there is a relationship between the value systems of pet owners and their presentation of animals for sterilisation (Smith 2014). In fact, studies in Romania have revealed that men were less inclined than women to have their dogs castrated; however, no gender bias was found in the way both sexes disapproved of spaying (Cocia & Rusu 2015). Thus, cultural norms, social influences, individual attitudes and economic considerations of Namibia’s underserved communities might have influenced the findings of this study.

Overall, a greater proportion of the presented pets were owned by men than by women. In addition, a greater proportion of male pets were male-owned than female-owned (34.3% and 29.6%, respectively). There are many reports advocating that sterilisation of pets not needed for breeding is an indicator of responsible pet ownership. Studies have demonstrated that individual attitudes, cultural norms, social norms, economic considerations, location (rural or urban) and owner perceptions of pets all have a bearing on pet owners’ support for sterilisation (Downes et al. 2015; Murray et al. 2015). Men encountered in this study presented more dogs than cats for sterilisation (55.5% and 8.5%, respectively). Similarly, women also presented more dogs than cats for sterilisation (28.9% and 7.1%, respectively).

The observation that men present more male dogs for sterilisation is consistent with a study from Romania (Cocia & Rusu 2010). However, in this Romanian study, men disapproved of sterilisation of male pets, whilst women tended to approve of the same, and there was no owner gender bias when it came to sterilisation of female pets (Cocia & Rusu 2010). According to Cocia and Rusu (2010), the gender bias in approving or disapproving of sterilisation of specific pet genders was related to anthropological, sociological and evolutionary considerations and could be a consequence of historical gender conflict promoted in Romania during the Soviet era. This study, however, did not reflect the women’s support for male sterilisation. The observation of female owners supporting female cat sterilisation more than male cats has not been reported previously.

The results of this study show that significantly more pets were presented to the mobile clinic for sterilisation from urban than from rural communities (68.2% and 31.8%, respectively). Overall, in urban areas, a greater proportion of men than women presented pets for sterilisation (43.8% and 24.4%, respectively); however, in rural areas, a greater proportion of women than men presented pets for sterilisation (20.1% and 11.7%, respectively). Although, overall, more dogs than cats were presented for sterilisation (84.4% and 15.6%, respectively), a greater proportion of cats were presented in the urban communities than in the rural communities (11.7% and 3.9% respectively). Ortega-Pacheco et al. (2007) also reported more dogs presented for sterilisation in urban centres than in rural centres in Yucatan. In this study, higher numbers of pets were presented in smaller urban centres of Rundu and Luderitz. This observed difference could be because of the fact that this study only targeted underserved communities or because there being more poor people in smaller than in larger urban centres of Namibia.

Other studies have reported entirely different results in which more dogs than cats were presented for sterilisation in rural centres (Acosta-Jamett et al. 2010; Burns 2019; Carvelli et al. 2016; Knobel et al. 2008). Rural settings in the developed world (Italy) may not necessarily be comparable with the rural settings of the developing world, and this could explain disparity in cat ownership between the two studies (Carvelli et al. 2016).

It is noteworthy that a high proportion of dogs in underserved communities are sterilised only after 2 years. The delayed sterilisation could be related to inability of pet owners in these communities to afford the cost of sterilisation. The mobile clinic team in this study always made sure that they revisited locations every 6 months so as to sterilise pets aged <6 months or 6–8 months as well as those born soon after the preceding visit before they attain puberty.

The owners of the dogs and cats in this study were informed about the benefits of sterilisation of pets and the primary healthcare that was associated with the procedures before they volunteered their pets for sterilisation. It has been pointed out that the cost of surgery is a substantive issue about owners’ willingness to present their pets for sterilisation (Faver 2009). Hence, the BAINES VETCARE/SoVM Mobile Clinic’s free sterilisation of pets as a community service is improving the coverage of sterilisation in Namibia.

Most dogs had names, whilst quite a number of cats were not named. Globally (Borkfelt 2011; Kido 2017, 2018) as well as in the African continent (Olaosun & Arua 2012), limited studies have reported on the patterns of nomenclature of pets. Analysis of pet names lends credence to linguistic (Kido 2017, 2018) and psychological semiotic discourse (Olaosun & Arua 2012) or both (Borkfelt 2011). Taking the linguistic route, results of this study revealed that there were major language differences amongst Namibia’s 13 ethnic groups, although commonalities existed between some groups of vernacular African languages. Some similarities existed within both Bantu and San languages. Taking the semiotic route, there were similarities in values across ethnic groups. In some cases, the names of pets reflected the owner’s level of education or the pet’s behaviour. For instance, some names took thematic and attributive roles, describing the personal circumstances of the owners, and yet others were satirical or metaphorical. Results from this study were similar to those obtained by Olaosun and Arua (2012), who studied the semiology of pet naming in Yoruba and Igbo cultures of Nigeria.

Thus, it is concluded that the campaign to sterilise pets in the underserved communities of Namibia by the BAINES VETCARE/SoVM Mobile Clinic as a community engagement exercise is an important step towards pet health management and population control. The exercise inherently captures important background epidemiological information on the structure of pet populations, socio-economic information of their owners and information on the relationship between owners and their pets. Such information is critical for the surveillance and control of zoonoses as well as for the evaluation of interventions.
